# The first metatarsophalangeal joint in gout: a systematic review and meta-analysis

**DOI:** 10.1186/s12891-016-0919-9

**Published:** 2016-02-11

**Authors:** Sarah Stewart, Nicola Dalbeth, Alain C. Vandal, Keith Rome

**Affiliations:** Faculty of Health and Environmental Sciences, Health & Rehabilitation Research Institute, Auckland University of Technology, Private Bag 92006, Auckland, 1142 New Zealand; Department of Medicine, Faculty of Medical and Health Sciences, The University of Auckland, Private Bag 92019, Auckland, 1142 New Zealand; Department of Biostatistics & Epidemiology, School of Public Health & Psychosocial Studies, Faculty of Health and Environmental Sciences, Auckland University of Technology, Private Bag 92006, Auckland, 1142 New Zealand; Health Intelligence & Informatics, Ko Awatea, Counties Manukau Health, Private Bag 93311, Auckland, 1640 New Zealand

**Keywords:** First metatarsophalangeal joint, Gout

## Abstract

**Background:**

The aim of this review was to qualitatively synthesise studies that have investigated characteristics of the first metatarsophalangeal joint (1^st^ MTP) in gout and to undertake a meta-analysis to estimate the average prevalence of acute 1^st^ MTP arthritis across studies in people with gout.

**Methods:**

Studies published in English were included if they involved participants who had a diagnosis of gout and presented original findings relating to the following outcome measures associated with the 1^st^ MTP: epidemiology; clinical features; structural and functional characteristics; and microscopic and imaging features.

**Results:**

Forty-five studies were included in the qualitative synthesis. 1^st^ MTP pain was a prominent feature in people with gout. People with 1^st^ MTP gout reported walking- and general-disability. Structural and functional characteristics of 1^st^ MTP gout included hallux valgus, osteoarthritis, and restricted joint motion. Successful crystal aspiration ranged from 81 to 91 % and positive crystal identification via microscopy ranged from 83 to 93 % in patients with a history of 1^st^ MTP gout. Imaging features were common at the 1^st^ MTP including the double contour sign, tophi and erosions. Eleven studies involving 2,325 participants were included in the meta-analysis, providing an estimate of the average prevalence of acute 1^st^ MTP arthritis across studies of 73 % (95 % prediction interval 40–92 %; range 48–97 %; I^2^ = 93 %).

**Conclusions:**

1^st^ MTP acute arthritis is highly prevalent in people with gout and has a substantial impact on patient-reported pain and disability. Gout affects the structure and function of the 1^st^ MTP. Microscopic and imaging studies have demonstrated crystal deposition and joint damage at the 1^st^ MTP in people with gout.

## Background

Gout, one of the most common forms of inflammatory arthritis in middle-aged men, is caused by monosodium urate (MSU) crystal deposition in joints and soft tissue [1]. Clinically, gout is characterised by painful flares of acute monoarthritis interspersed with asymptomatic periods. If left untreated, gout can progress to a chronic arthritis with tophus formation and joint damage [2].

Gout is well recognised by its predilection to affect the first metatarsophalangeal joint (1^st^ MTP). Acute episodes of gouty arthritis at the 1^st^ MTP are often referred to as *podagra* [3]. In recent decades, lifestyle and dietary factors associated with hyperuricaemia have become increasingly widespread, as has the global burden of gout [4]. Gout has a major impact on health-related quality of life [5] and its tendency to affect the foot is reflected in the high levels of foot-related disability and impairment [6].

Despite the well-recognised susceptibility of the 1^st^ MTP to acute arthritis in gout, evident by its inclusion in several gout diagnostic and classification criteria [7–10], a formal synthesis of the prevalence of acute 1^st^ MTP arthritis in this condition has yet to be undertaken. Furthermore, the burden of 1^st^ MTP involvement on patient-reported outcomes in gout is unclear. Despite the significant role of the 1^st^ MTP during normal gait, particularly the forward transfer of body weight during propulsion [11, 12], it is also unclear to what extent the structure and function of the joint is compromised in people with gout. This systematic review aimed to qualitatively synthesise studies which have investigated characteristics of the 1^st^ MTP in gout and to undertake a meta-analysis to provide a pooled estimate for the average prevalence of acute 1^st^ MTP arthritis in gout across studies.

## Methods

### Search strategy

A comprehensive electronic search was completed in March 2015 using the following databases: Scopus (1960 to March 2015), Medline (1966 to March 2015), CINAHL (1937 to March 2015), SportsDiscus (1985 to March 2015), the Cochrane Library, ACR abstracts (2009 to 2013) and EULAR abstracts (2002 to 2012) with the search terms presented in Table 1. This search was supplemented with hand-searching of reference lists of all potentially eligible full-text articles and selected review articles.

**Table 1:** Search terms

#1	Gout* OR uric OR urate OR hyperuric*OR toph*
#2	“First metatarsophalangeal” OR “first metatarsal phalangeal” OR hallux OR “big toe” OR “first toe” OR digit OR podagra OR “1st mtp*” OR 1mtp* OR foot OR feet
#3	Imaging OR sonograph* OR ultraso* OR “doppler” OR radiograph* OR xray OR “magnetic resonance” OR mri OR “computed tomograph*” OR ct OR “dect” OR “dual energy”
#4	Histol* OR microscop*
#5	Function* OR gait OR walk* OR “plantar pressure” OR motion

Final search term: #1 AND #2 AND (#3 OR #4 OR #5)

(gout* OR uric OR urate OR hyperuric*OR toph*) AND (“first metatarsophalangeal” OR “first metatarsal phalangeal” OR hallux OR “big toe” OR “first toe” OR digit OR podagra OR “1st mtp*” OR 1mtp* OR foot OR feet) AND ((imaging OR sonograph* OR ultraso* OR “doppler” OR radiograph* OR xray OR “magnetic resonance” OR mri OR “computed tomograph*” OR ct OR “dect” OR “dual energy”) OR (histol* OR microscop*) OR (function* OR gait OR walk* OR “plantar pressure” OR motion))

### Selection criteria

All potentially eligible articles were screened by a single author (SS) at title, abstract, and full-text stages. The review was conducted with reference to the Preferred Reporting Items for Systematic review and Meta-Analysis protocols (PRISMA) statement [13]. Studies considered for this review were published in peer-reviewed journals and limited to randomised controlled trials, cohort studies, case-control studies and cross-sectional studies. Peer-reviewed conference proceedings and abstracts were also considered for inclusion. Case reports, case series with <5 cases and review articles were excluded.

The inclusion criteria included studies published in English, adults over 18 years old and which involved participants who had a diagnosis of gout. Included studies presented original findings relating to the following outcome measures: incidence or prevalence of acute inflammatory arthritis at the 1^st^ MTP; clinical features of acute gouty arthritis, intercritical gout and chronic gouty arthropathy (including tophaceous gout) at the 1^st^ MTP; structural and functional characteristics of the 1^st^ MTP; microscopy of the 1^st^ MTP; and imaging features of the 1^st^ MTP including MSU crystals, bone disease and synovial disease. Studies investigating outcomes as a measure of pharmacological, non-pharmacological and surgical intervention efficacy were excluded. Studies which assessed the 1^st^ MTP amongst other joints, but did not report outcome measures specifically relating to the 1^st^ MTP, were also excluded.

### Data extraction

The following data was extracted from all included papers: the first author’s last name, publication year, country where the study was conducted, the study design and aim(s), the outcome measure(s) reported and the characteristics of the gout participants including: sample size, gender, mean age (years), mean disease duration (years) and the method of diagnosis.

### Statistical analysis

A meta-analysis was conducted to obtain an estimate of the prevalence of acute arthritis at the 1^st^ MTP in people with gout at any point during the course of their disease. Due to the expected high prevalence of acute 1^st^ MTP arthritis, a double arcsine transformation was adopted to address variance instability. This transformation method is the preferred transformation option as it avoids an undue large weight for studies [14]. The meta-analysis was carried out using the inverse of the variance of the transformed proportion as study weight. The pooled transformed prevalence was transformed back for the final presentation of the data. The 95 % prediction intervals for the average estimate of prevalence was also reported [15]. A random-effects model was used and the degree of heterogeneity was evaluated using the Higgins I^2^ statistic which was interpreted as follows: I^2^ of 25 % = low heterogeneity, I^2^ of 50 % = medium heterogeneity, I^2^ = 75 % = high heterogeneity [16]. Statistical analysis was undertaken in MetaXL version 2.0 (EpiGear International Pty Ltd, Brisbane, Australia).

## Results

### Description of studies

**Fig. 1:**
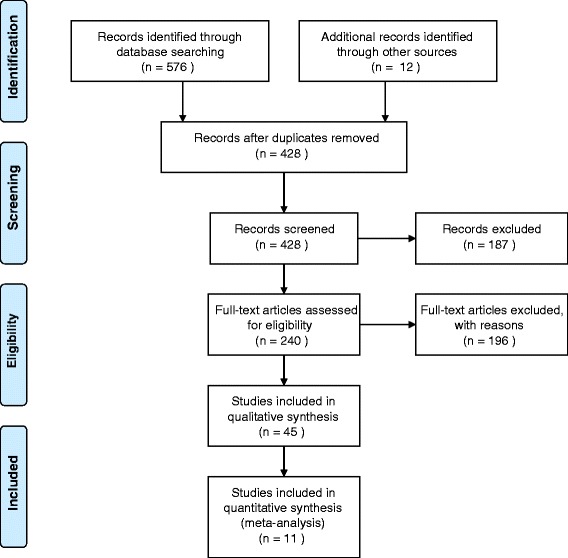
PRISMA flowchart

Figure 1 shows a flow chart of the literature search. The initial search identified 576 papers through database searching and 12 papers from conference abstracts. Following the removal of 160 duplicates, 428 papers were screened, of which 240 papers were considered for further examination based upon the title and abstract. Forty-five studies met the criteria and were included in the review (including 4 conference abstracts published in peer-reviewed journals [17–20] and 2 English abstracts from non-English papers [21, 22]). Of the 45 studies, 8 were longitudinal cohort studies, 20 cross-sectional studies, 10 case-control studies, 5 retrospective studies, and 2 randomised clinical trials. Details of the 45 included studies are displayed in Table 2.

**Table 2:** Details of papers included in review

Author	Location	Study design	Aim	Characteristics of gout participants	Outcome measures relevant to review
Bellamy [27]	Canada	Longitudinal cohort	Natural progression of 1MTP acute flares	*N* = 11 Male 100 % Mean age 55 years Mean disease duration 4 years Diagnosis: “patients presenting with classical features of acute podagra, had experienced prior attacks of gouty arthritis and were known to be or have been hyperuricaemic”.	Clinical characteristics of 1MTP gout
Carter [32]	USA	Cross-sectional	The presence of synovial inflammation during intercritical gout	*N* = 72 Male 90 % Mean age 56 years Mean disease duration 10 years Diagnosis: ACR criteria (incl. 42 % crystal proven)	Epidemiology of 1MTP gout
Dalbeth [58]	New Zealand	Cross-sectional	Scoring bone erosion in gout using CT imaging	*N* = 25 Male 75 % Median age 60 years Median disease duration 21 years Diagnosis: ACR criteria (incl. 44 % crystal proven)	Imaging features of the 1MTP in gout
Dalbeth [55]	New Zealand	Cross-sectional	Relationship between radiographic joint damage and MSU crystal deposition using DECT imaging	*N* = 92 Male 93 % Mean age 58 years Mean disease duration 22 years Diagnosis: ACR criteria (incl. 48 % crystal proven)	Imaging features of the 1MTP in gout
Dalbeth [54]	New Zealand	Cross-sectional	Comparison of DECT MSU deposition in people with gout and people with asymptomatic hyperuricaemia	*N* = 33 Male = 85 % Mean age 61 years Mean disease duration 11 years Diagnosis: MSU crystal proven	Imaging features of the 1MTP in gout
Deesomchok [34]*	Thailand	Cross-sectional	Clinical pattern of gout in females and males	*N* = 194 Male 89 % Mean age 59 (F) 52 (M) years Mean disease duration 3 (F) 10 (M) years Diagnosis: ACR criteria (incl. 81 % crystal proven)	Epidemiology of 1MTP gout
DeSouza [36]	Brazil	Cross-sectional	Clinical and laboratory features of gout in men and women	*N* = 58 Male 53 % Mean age 64 (F) 61 (M) years Mean disease duration 9 (F) 14 (M) years Diagnosis: ACR criteria (% crystal proven not reported)	Epidemiology of 1MTP gout
Grahame [29]*	UK	Retrospective	Characteristics of patients with gout	*N* = 354 Male 90 % Mean age NR Mean disease duration NR Diagnosis: patients with “recurrent acute episodes of arthritis in the presence of hyperuricaemia”	Epidemiology of 1MTP gout
Hall [28]*	USA	Longitudinal cohort	Epidemiology of gout and hyperuricaemia	*N* = 86 Male 87 % Mean age 58 years Mean disease duration 9 years Diagnosis: patients who met two of the following: a typical attack of arthritis characterised by acute pain, usually accompanied by swelling and heat, lasting from a few day sot 2 weeks, and followed by complete remission of symptoms; an attack of arthritis exhibiting a striking and prompt response to therapeutic colchicine; the presence of hyperuricaemia.	Epidemiology of 1MTP gout
Howard [67]	USA	Cross-sectional	Reliability of ultrasound for tophi and double contour sign	*N* = 50 Male 100 % Mean age 69 years Mean disease duration NR Diagnosis: ACR criteria (% crystal proven not reported)	Imaging features of the 1MTP in gout
Huppertz [31]*	Germany	Case-control	Diagnostic accuracy of DECT and ultrasound for detecting MSU crystal deposition	*N* = 39 Male NR Mean age NR Disease duration newly diagnosed Diagnosis: Janssens score [10] of >8 or MSU crystal proven (46 %)	Imaging features of the 1MTP in gout
Janssens [10]*	Netherlands	Cross-sectional	Validation of a diagnostic model to predict gout	*N* = 209 Male 89.5 % Mean age 59 years Mean disease duration NR Diagnosis: MSU crystal proven	Epidemiology of 1MTP gout
Kawenoki-Minc [21]	Poland	Cross-sectional	Incidence of radiographic evidence of degenerative articular changes in patients with gout	*N* = 262 Male NR Mean age NR Mean disease duration NR Diagnosis: not reported	Imaging features of the 1MTP in gout
Kennedy [30]	London	Cross-sectional	MSU crystal presence in aspirated 1MTP joint fluid	*N* = 31 Male 100 % Mean age 61 years Mean disease duration 10 years Diagnosis: patients who had “previously had podagra” including 52 % who were MSU crystal proven	Microscopy in 1MTP gout
Kienhorst [37]	Netherlands	Longitudinal cohort	Validation of clinical diagnosis of 1MTP gout	*N* = 123 Male 85 % Mean age 59 years Mean disease duration NR Diagnosis: MSU crystal proven	Clinical characteristics of 1MTP gout
Kim [68]	Korea	Cross-sectional	DECT presence of MSU deposition	*N* = 101 Male 95 % Mean age NR Mean disease duration NR Diagnosis: ACR criteria (% crystal proven not reported)	Imaging features of the 1MTP in gout
Kim [43]	Korea	Longitudinal cohort	Comparison of clinical outcomes of arthrodesis and tophi excision & evaluate MRI features of tophaceous gout	*N* = 15 Male 67 % Mean age 57 years Mean disease duration NR Diagnosis: MSU crystal proven	Clinical characteristics of 1MTP gout; Functional characteristics of 1MTP gout; Imaging features of the 1MTP in gout
Lally [35]*	USA	Retrospective	Comparison of gouty arthritis in men and women	*N* = 98 Male 77 % Mean age 63 (F) 63 (M) years Mean disease duration 5 (F) 14 (M) years Diagnosis: MSU crystal proven	Epidemiology of 1MTP gout
Mallinson [25]	Canada	Retrospective	Distribution of MSU crystal deposition in gout using DECT imaging	*N* = 148 Male 82 % Mean age 61 years Mean disease duration NR Diagnosis: not reported	Microscopy in 1MTP gout
Mijiyawa [38]*	Togo	Retrospective	Characteristics of patients with gout	*N* = 106 Male 99 % Mean age 53 years Mean disease duration 8 years Diagnosis: ACR criteria (incl. 17 % crystal proven)	Epidemiology of 1MTP gout
Kang [41]	Korea	Longitudinal cohort	Ultrasound characteristics of gout & efficacy of intra-articular steroid injection for acute 1MTP flares	*N* = 21 Male 86 % Mean age 64 years Mean disease duration NR Diagnosis: ACR criteria (% crystal proven not reported)	Clinical characteristics of 1MTP gout; Imaging features of the 1MTP in gout
Naredo [50]	Spain	Case-control	Diagnostic value of ultrasound for gout	*N* = 91 Male 100 % Mean age 56 years Mean disease duration 7 years Diagnosis: MSU crystal proven	Imaging features of the 1MTP in gout
Ordonez [17]	Spain	Cross-sectional	Reliability of ultrasound in detection of lesion at 1MTP in gout	*N* = 15 Male NR Mean age NR Mean disease duration NR Diagnosis: not reported	Imaging features of the 1MTP in gout
Ottaviani [52]	France	Longitudinal cohort	Ultrasonography showing disappearance of MSU crystals following urate lowering therapy	*N* = 16 Male 100 % Mean age 61 years Mean disease duration 7 years Diagnosis: MSU crystal proven	Imaging features of the 1MTP in gout
Pascual [46]	Spain	Cross-sectional	MSU crystal presence in aspirated 1MTP joint fluid	*N* = 101 Male 98 % Mean age 56 years Mean disease duration 5 years Diagnosis: ACR criteria (incl. 77 % crystal proven)	Microscopy in 1MTP gout
Pascual [69]	Spain	Longitudinal cohort	Disappearance of MSU crystals following urate lowering therapy	*N* = 18 Male 100 % Mean age 56 years Mean disease duration 10 years Diagnosis: MSU crystal proven	Microscopy in 1MTP gout
Pelaez-Ballestas [9]*	Mexico	Cross-sectional	Identifying criteria for diagnosis of chronic gout	*N* = 549 Male 96 % Mean age 50 years Mean disease duration 12 years Diagnosis: MSU crystal proven	Epidemiology of 1MTP gout
Radak-Perovic [22]	Serbia	Case-control	Comparison of ultrasound and x-ray for detection of 1MTP erosions	*N* = 30 Male NR Mean age NR Mean disease duration NR Diagnosis: ACR criteria (% crystal proven not reported)	Imaging features of the 1MTP in gout
Roddy [24]	UK	Case-control	Concomitant gout and osteoarthritis	*N* = 164 Male 81 % Mean age 63 years Mean disease duration 10 years Diagnosis: 91 % met ACR criteria (% crystal proven not reported)	Epidemiology of 1MTP gout; Functional characteristics of 1MTP gout
Roddy [51]	UK	Cross-sectional	Ultrasound characteristics of gout	*N* = 40 Male 78 % Mean age 65 years Mean disease duration 13 years Diagnosis: ACR criteria (incl. 53 % crystal proven)	Imaging features of the 1MTP in gout
Roddy [18]	UK	Cross-sectional	Prevalence of hallux valgus, foot pain and disability in gout	*N* = 1184 Male 78 % Mean age 66 years Mean disease duration 12 years Diagnosis: “Patients who had consulted their GP about gout or been prescribed allopurinol or colchicine within the preceding 2 years”	Clinical characteristics of 1MTP gout; Structural characteristics of 1MTP gout
Roddy [23]	UK	Case-Control	Nodal osteoarthritis and the risk of developing gout	*N* = 164 Male 81 % Mean age 63 years Mean disease duration 10 years Diagnosis: 91 % met ACR criteria (% crystal proven not reported)	Clinical characteristics of 1MTP gout; Structural characteristics of 1MTP gout
Rome [45]	New Zealand	Case-control	Functional and biomechanical characteristics of gait in people with gout	*N* =25 Male 75 % Mean age 61 years Mean disease duration 22 years Diagnosis: ACR criteria (incl. 44 % crystal proven)	Functional characteristics of 1MTP gout
Rouault [39]*	USA	Case-control	1MTP aspiration as a diagnostic tool for gout	*N* = 23 Male NR Mean age NR Mean disease duration NR Diagnosis: MSU crystal proven	Epidemiology of 1MTP gout; Microscopy in 1MTP gout
Sivera [47]	Spain	Cross-sectional	Feasibility of 1MTP aspiration for gout diagnosis	*N* = 22 Male 82 % Mean age 61 years Mean disease duration NR Diagnosis: MSU crystal proven	Microscopy in 1MTP gout
Sun [53]	China	Cross-sectional	DECT imaging features of gout	*N* = 80 Male 94 % Mean age 52 years Mean disease duration 5 years Diagnosis: ACR criteria (% crystal proven not reported)	Imaging features of the 1MTP in gout
Taylor [33]*	Multinational, multicentre	Cross-sectional	Clinical, laboratory, imaging findings in gout	*N* = 509 Male 86 % Mean age 60 years Median disease duration 6 years Diagnosis: MSU crystal proven	Epidemiology of 1MTP gout; Clinical characteristics of 1MTP gout
Thiele [56]	USA	Case-control	Sonographic features of gout	*N* = 16 Male 81 % Mean age 64 years Mean disease duration NR Diagnosis: MSU crystal proven	Imaging features of the 1MTP in gout
Thiele [57]	USA	Retrospective	Detection of erosions with ultrasound and conventional radiography in chronic gout	*N* = 42 Male NR Mean age NR Mean disease duration NR Diagnosis: MSU crystal proven	Imaging features of the 1MTP in gout
Vreju [19]	Romania	Longitudinal cohort	Importance of ultrasound in gout diagnosis	*N* = 23 Male 53 % Mean age 53 years Disease duration < 1 year Diagnosis: not reported	Imaging features of the 1MTP in gout
Wang [26]	Taiwan	Randomised controlled trial	Comparison of arthroscopic removal of 1MTP MSU crystals with medical treatment alone	*N* = 28 Male 100 % Mean age 28 years Mean disease duration 2 years Diagnosis: not reported	Clinical characteristics of 1MTP gout; Functional characteristics of 1MTP gout
Wallace [7]*	USA	Cross-sectional	Development of a criteria for the classification of arthritis due to primary gout	*N* = 178 Male 86 % Mean age 56 years Mean disease duration 10 years Diagnosis: 88 % met ACR criteria (incl. 43 % crystal proven)	Epidemiology of 1MTP gout
Weinberger [48]	USA	Case-control	MSU crystals in aspirated 1MTP joint fluid	*N* = 9 Male 100 % Mean age 54 years Mean disease duration 2 years Diagnosis: MSU crystal proven	Microscopy in 1MTP gout
Wright [42]	Ireland	Case-control	Comparison of ultrasound and x-ray for detection of erosions in gout	*N* = 39 Male 100 % Mean age 52 years Mean disease duration 12 years Diagnosis: ACR criteria (incl. 28 % crystal proven)	Imaging features of the 1MTP in gout
Zleik [20]	USA	Longitudinal cohort	Risk & predictors additional flares in newly diagnosed gout	*N* = 158 Male 73 % Mean age 59 years Mean disease duration Newly diagnosed Diagnosis: via the New York, Rome or ACR criteria (% crystal proven not reported)	Epidemiology of 1MTP gout

*Papers included in meta-analysis; NR = Not Reported

The 45 included studies involved 44 different groups of gout participants (two studies used the same participants [23, 24]) totalling a pooled sample size of 5,478 participants. Thirty-eight studies involving 5,067 participants reported gender of which 4,348 (86 %) were male. Thirty-six studies reported mean participant age which ranged from 28 years to 69 years. Twenty-nine studies reported disease duration which ranged from newly diagnosed gout to 22 years.

Five studies did not report how patients with gout were diagnosed [17, 19, 21, 25, 26]. Fifteen studies, totalling 1,773 participants included only patients with gout who were diagnosed via microscopic identification of MSU crystals in synovial fluid/tophus aspirates. Fifteen studies, totalling 1,116 participants, diagnosed gout via the 1977 ACR criteria [7] in which participants either had MSU-proven gout or met 6 of the 12 clinical criteria. Of these studies, nine reported the number of crystal-proven participants (300/656 (46 %)). Two of these studies knowingly included patients with gout who did not meet the ACR criteria (*n* = 36). The remaining seven studies included in the review diagnosed patients with gout using other methods detailed in Table 2 [18, 20, 27–31].

The 45 studies reported on one or more of the following outcome measures relating to 1^st^ MTP gout: epidemiology (*n* = 14, including *n* = 11 articles reporting on the prevalence of acute 1^st^ MTP arthritis at any point during the course of the disease which were included in the meta-analysis), clinical characteristics (*n* = 8), structural characteristics (*n* = 2), functional characteristics (*n* = 4), microscopy (*n* = 7); and imaging features (*n* = 19).

### Epidemiology

Acute 1^st^ MTP arthritis presenting as the manifestation of gout at disease onset, ranged from 43 to 76 % [20, 32–35]. The frequency of acute 1^st^ MTP arthritis as the initial manifestation of gout was not significantly different between genders [34–36]. However, acute 1^st^ MTP arthritis at any point during the disease was significantly more frequent in men (68.6 %) compared to women (31.8 %) [34, 36]. Two studies reported 54 and 72 % of patients with gout, respectively, experienced acute arthritis isolated to the 1^st^ MTP [33, 37].

**Fig. 2:**
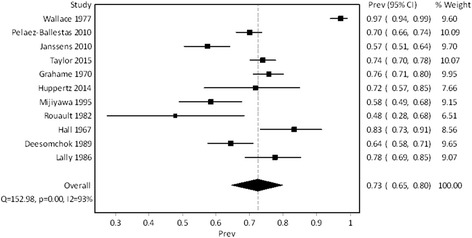
Forest plot showing pooled study prevalence of acute 1st MTP arthritis in gout

Eleven studies reported the prevalence of acute 1^st^ MTP arthritis at any point during the course of the disease and were included in the meta-analysis [7, 9, 10, 28, 29, 31, 33–35, 38, 39]. The studies provided a pooled sample size of 2,464 participants. In total, 87 % were male with a mean age ranging between 50 and 63 years old. Mean or median disease duration was reported by eight studies and ranged from newly diagnosed to 14 years [7, 9, 28, 31, 33–35, 38]. Fifty-six percent of 2,110 participants from 10 studies demonstrated aspirate proven gout [7, 9, 10, 28, 31, 33–35, 38, 39]. The reported prevalence of acute arthritis at any point during the course of the disease ranged from 48 to 97 %. The pooled prevalence estimate of acute 1^st^ MTP arthritis across studies was 73 % (95 % prediction interval: 40–92 %). The heterogeneity was high with an I^2^ of 93 % (95 % CI: 90 %–96 %). Figure 2 presents the forest plot showing the pooled prevalence estimate of acute 1^st^ MTP arthritis in gout across the included studies.

### Clinical characteristics

Four studies reported the characteristics of acute 1^st^ MTP arthritis, which included rapid onset of extremely severe pain and tenderness with moderate swelling, erythema and inflammation [27, 37, 40]. Seventy-nine percent of patients reported onset within 1 day [37]. Erythema was observed in 95 % of patients with acute 1^st^ MTP arthritis [37]. In a study following the natural progression of acute 1^st^ MTP arthritis for 7 days in 11 patients, improvements in erythema and juxta-articular skin temperature were observed by day four, while 1^st^ MTP pain and swelling improved in most by day five [27]. Two studies reported pain during acute 1^st^ MTP arthritis using 100 mm Visual Analogue Scales (VAS) [37, 41]. Mean patient-reported pain ranged from 54.3 to 71.1 mm [37, 41].

During intercritical periods, in the absence of acute symptoms, a significantly greater number of patients with gout reported current 1^st^ MTP pain compared to healthy matched controls (16 % vs. 6 %, respectively) [23]. One study reported 72 % of patients with long-standing gout, with a mean of 12 years disease duration, reported 1^st^ MTP pain in the previous month [18]. During clinical examination of 78 1^st^ MTPs from 39 patients with currently asymptomatic gout, 35 % of joints demonstrated mild tenderness, 9 % demonstrated moderate tenderness and 6 % demonstrated marked tenderness [42].

High levels of 1^st^ MTP pain were reported in patients with chronic tophaceous gout affecting the 1^st^ MTP [26, 43], with patients scoring a mean of 7.6 cm to 7.8 cm on a 10 cm pain VAS [43].

### Structural characteristics

Structure of the 1^st^ MTP has also been assessed in people with gout through the presence of self-reported hallux valgus, a structural forefoot deformity involving lateral deviation of the hallux at the 1^st^ MTP [18, 23]. A case-control study involving 164 patients with gout found that self-reported hallux valgus was significantly more common in patients with gout [23]. However, a larger cohort study involving 1,184 gout participants revealed 36 % of patients had self-reported hallux valgus, which the authors reported was similar to the general population and not related to gout-specific factors [18].

### Functional characteristics

Moderate to high levels of general disability and walking disability were reported using 100 mm VAS in patients with current acute 1^st^ MTP arthritis (mean 60.0 mm to 64.1 mm) [41]. Similarly, when using the Hallux Metatarsophalangeal-Interphalangeal section of the American Orthopaedic Foot and Ankle Society (AOFAS) scale [44], which provides a total score out of 100 based on pain, range of motion, joint instability and alignment, and activity- and footwear-related limitations, people with 1^st^ MTP tophi scored between 65 and 61 out of 100, where 100 indicates an absence of pain and any joint, activity or footwear limitation [43].

A study on 164 people with gout found a significant association between acute 1^st^ MTP arthritis and the presence 1^st^ MTP osteoarthritis, defined as restricted motion, bony swelling and/or crepitus [24]. In a further study of patients with severe tophaceous gout the mean (SD) total range of motion at the 1^st^ MTP was 19°, which reduced to 14° in joints which also demonstrated severe cartilage loss [43].

A study assessing foot function in 25 patients with long-standing chronic gouty arthropathy using in-shoe plantar pressure analysis found peak plantar pressure beneath the first metatarsal was greater in the gout group, although not significantly [45]. However, peak plantar pressure and pressure-time integrals beneath the hallux were significantly reduced in the gout group when compared to the controls, which the authors proposed reflected an attempt to offload pain at the 1^st^ MTP [45].

### Microscopy

The success of joint fluid acquisition for the purposes of microscopic identification of MSU crystals from the 1^st^ MTP ranged from 81 to 91 % [30, 46, 47]. The presence of MSU crystals in 1^st^ MTP fluid ranged from 83 to 89 % of currently asymptomatic 1^st^ MTPs in patients with gout with a history of acute 1st MTP arthritis [39, 46]. In patients with gout with no history of acute 1st MTP arthritis, 52 to 67 % of aspirated 1st MTPs were positive for crystals [30, 39, 48]. In patients experiencing current acute 1st MTP arthritis the presence of MSU crystals ranged from 85 to 93 % [47, 49].

The occurrence of acute 1st MTP arthritis as a diagnostic feature has been compared with the presence of MSU crystals in aspirated 1st MTP fluid [10, 37]. Based on clinical characteristics of acute 1st MTP arthritis in 159 patients, general practitioners diagnosed 98 % of patients as having gout [37]. When validated against the presence of MSU crystals, a sensitivity of 0.99 and a specificity of 0.08 was demonstrated [37]. In a study of 328 patients with monoarthritis, Janssens [10] reported that the 1st MTP as the location of the monoarthritis was an independent predictor of MSU crystal presence [10].

### Imaging features

#### Urate crystal deposition

Ultrasonography allows the visualisation of MSU crystal deposition along the surface of articular cartilage, referred to as the ‘double contour sign’, the presence of which ranged from 22 to 87.5 % at the 1st MTP [41, 42, 50–52]. Naredo [50] noted the double contour sign was more frequent at the dorsal aspect of the 1st MTP (62 %) compared to the plantar aspect (23 %).

Dual-energy computed tomography (DECT) may be less sensitive than ultrasound due to the lower spatial resolution [31]. In a study of 39 patients (79 % with newly diagnosed gout) DECT detected urate crystals in 26 % of 1st MTPs, and ultrasound in 74 % of the same joints [31]. The presence of MSU deposition using DECT increased from 26 % of joints in newly diagnosed gout [25], to 36 % after 5 years disease duration [53], and 54 % in patients with 11 years mean duration [54]. In patients with tophaceous gout and a mean disease duration of 22 years, MSU crystals were present in 38 % of 184 1st MTPs [55].

In a DECT study assessing feet with current flares, the authors reported crystal deposition in 41 % of 1st MTPs in patients with 1st MTP flares, compared to 27 % of 1st MTPs in patients with current ankle flares [53]. The presence of DECT MSU crystal deposition was found to be a risk factor for acute 1st MTP arthritis (OR = 3.38;) [53].

#### Tophi

In sonographic studies, the presence of tophi in 1st MTPs of people with gout ranged from 50 to 100 % [41, 42, 50, 52, 56, 57]. Thiele and Schlesinger [56] noted tophi were more often seen medial and dorsal to the joint with a distinct pattern in which unformed micro-particles were seen in the dorsal proximal recess and central area while formed tophi were more frequent in the medial compartment and impinging on the dorsal proximal phalanx [56]. Using MRI, a study of 15 patients with 1st MTP tophaceous gout, reported that the medial sesamoid was the most common location for tophaceous infiltration (seen in 47 % of patients), followed by the first metatarsal shaft (40 %) and lateral sesamoid (33 %) [43]. In the majority of patients (57 %) tophi were observed both extra-articularly and intra-articularly within the 1st MTP.

#### Bone disease

In patients with long-standing tophaceous gout, erosions on conventional radiography were noted in 79 % of 1st MTPs [55]. Other radiographic features of bone damage have been observed frequently in the 1st MTP joints in people with gout including spur formation (40 % of joints), joint space narrowing (39 %), osteophytes (44 %), sclerosis (73 %) and periosteal new bone formation (13 %) [55]. A study of 262 patients with gout reported radiographic proven osteoarthritis (defined as destruction of the articular surfaces) in 44 % of 1st MTPs and found a significant correlation between osteoarthritis and acute arthritis at this joint [21].

Sonographic evidence of bone erosion in 1st MTPs ranged from 40 to 67 % in patients with gout [22, 41, 42, 56, 57]. Wright [42] reported 92 % of detected erosions were present on the medial aspect of the metatarsal head, with 7 % on the dorsal metatarsal head, and the remaining 2 % on the medial aspect of the proximal phalangeal base. Erosions at the 1st MTP can be multifocal or unifocal and generally measure at least 2 mm in diameter [41, 42]. Thiele and Schlesinger [56] noted all erosions at the 1st MTP were adjacent to tophaceous material.

Using MRI, Kim [43] reported erosions and intraosseous involvement present in the first metatarsal shaft of 40 % of patients with symptomatic 1st MTP tophaceous gout. Using conventional CT, Dalbeth [58] reported 78 % of 1st MTPs had erosions present at the first metatarsal head, and 34 % had erosions at the proximal phalanx. The proportions of eroded bone were also greater at the metatarsal head compared to the proximal phalanx and were higher in those with clinically-evident tophi.

#### Synovial disease

Synovial disease, in the form of joint effusion, synovial hypertrophy, and synovitis has been assessed using both gray-scale and power Doppler ultrasound [41, 42, 51, 56]. Joint effusion, which has been observed in 29 to 74 % of 1st MTPs in people with gout [41, 42, 51, 56], is less specific for gout and has been seen at a similar rate in other rheumatic conditions (64 to 73 %) [42, 56]. Similarly, synovial hypertrophy is seen at a similar rate in gouty 1st MTPs (53 to 87 %) [41, 42, 51], and other rheumatic conditions (64 %) [42]. Synovitis, which can be assessed using power Doppler ultrasound has been shown to be more sensitive than clinical assessment (18 % vs. 5 %, respectively) [51]. Synovitis has been reported to occur at the 1st MTP with a mild Doppler signal in 15 %, moderate signal in 18 % and marked signal in 10 % [42]. Synovitis occurs at a significantly greater frequency in 1st MTPs in those joints with acute arthritis, with Kang [41] reporting 95 % of 1st MTPs with acute arthritis demonstrated mild to moderate power Doppler signals. However, synovitis is not specific to gout and is seen in 18 to 50 % of other inflammatory joint diseases [42, 56].

## Discussion

The historical observation of gout as a condition specifically affecting the 1st MTP is reflected in modern epidemiological literature, and is evident by the pooled 73 % prevalence estimate of 1st MTP acute arthritis reported in the meta-analysis of 11 studies. The clinical diversity between these studies, which is generally considered inevitable in meta-analyses [59], may explain the wide range of estimated prevalence values and account for the high heterogeneity observed. The included studies represented patients with gout from a wide range of countries, resulting in different participant demographics, genetic factors and lifestyle factors. Additionally, disease duration of gout participants varied considerably and is likely to impact the calculated prevalence estimate, as longer disease duration would increase the likelihood of experiencing an episode of acute 1st MTP arthritis. Furthermore, only 42 % of participants included in the meta-analysis were diagnosed with gout using the gold standard MSU crystal identification [7, 9, 10, 28, 31, 34, 35, 38, 39]. The differences in study designs adopted by the included studies (e.g. cohort, cross-sectional, case-control, retrospective and randomised controlled trial) may also have contributed to the increased heterogeneity. Nevertheless, this prevalence estimate provides useful quantitative data which corroborates the traditional notion that gout is a condition with frequent manifestations at the 1^st^ MTP.

Pain experienced during acute 1st MTP arthritis is considerable [27, 37, 41], and remains present following the resolution of acute symptoms [18, 42]. Outcome measurement methods used for measuring 1st MTP pain varied from 5-point Likert scales [27], visual analogue scales [37, 41], simply recording whether 1st MTP pain was present or absent [23], to measuring tenderness with palpation [42]. However, it appears that 1st MTP pain is a chronic foot problem in people with gout, which is further reflected by the sub-clinical joint inflammation observed during intercritical periods [42, 51]. In patients with 1st MTP tophaceous gout, high levels of pain are coupled with reduced joint function [26, 43]. Although it is unclear whether these clinical symptoms are a consequence of pain-avoidance, joint damage, synovial inflammation, mechanical obstruction by tophi, or a combination of these factors, the clinical implications of symptomatic 1st MTP gout on the ability to undertake everyday weight-bearing activities, such as walking, are recognised as important features of the disease [41]. People with gout walk significantly slower and demonstrate gait patterns consistent with 1st MTP pain-avoidance strategies [45]. Abnormal 1st MTP loading at toe-off in patients with gout may be further exacerbated by biomechanical strain as a result of MSU deposition within the 1st MTP flexor and extensor tendons [53, 60].

As the initiation of acute gouty arthritis is not possible in the absence of MSU crystals, the susceptibility of the 1st MTP to gout over other joints must be related to certain factors which predispose to the precipitation and deposition of crystals at this site. It has been hypothesised that the predilection for MSU deposition and patient symptoms in the foot and ankle may be attributed to the biomechanical loading or physical stress during the normal gait cycle [55, 56, 58, 60]. This is further emphasised in the distinct pattern of crystal deposition at the 1st MTP observed in imaging studies where MSU deposits have been reported to occur more often on the medial and dorsal aspects of the joint compared to the plantar aspect [50, 56, 61]. It has been proposed that this distinct pattern of crystal deposition at the 1st MTP may result from the shifting of tophaceous deposits with dorsiflexion during walking and their eventual clustering at pressure points within the joint [56].

Osteoarthritis observed at the 1st MTP has also been implicated in the co-occurrence of gout at this joint [21, 24]. However, the distinction between joint damage caused by chronic gouty arthritis and osteoarthritic joint damage is unclear, particularly due to the high prevalence of 1st MTP osteoarthritis in the general population [62].

This review has a number of limitations. Firstly, the literature search and screening was conducted by a single reviewer. Secondly, the methodology adopted may have created a selection bias through the exclusion of non-English language studies which may have resulted in an incompletely and potentially biased set of evidence. In regard to the methodologies used in included studies, most were cross-sectional descriptive studies which provide lower-level evidence and limit investigation in to the cause-effect relationship between 1st MTP characteristics and gout. Many of the studies also involved small sample sizes. Furthermore, gout disease characteristics of participants in the included studies varied and the majority of participants were diagnosed based on clinical criteria. Although this reflects diagnostic methods employed in clinical practice, there are several limitations to current classification criteria [40] and the demonstration of MSU crystals in synovial/tophus aspirates remains the only method to permit a definitive diagnosis of gout [63]. Lastly, there is an absence of a recommended outcome measure to assess patient-reported outcomes relating specifically to the 1st MTP in gout research which makes comparisons between studies challenging.

This review highlights the need for future research which adopts standardised assessment approaches when investigating patient-reported outcomes specifically relating to the 1st MTP in gout. Advanced imaging may be implemented to determine the structural characteristics of the joint in relation to clinical features, particularly how 1st MTP involvement affects patient-reported outcomes and the ability to carry out daily activities, including walking. This may direct further research which investigates the biomechanical role of the 1st MTP in the frequent occurrence of gout at this joint. By recognising the local factors that contribute to the 1st MTPs susceptibility to crystal deposition and inflammation, further studies may assess non-pharmacological interventions that are specifically aimed at the 1st MTP including footwear, foot orthoses and foot-related health education which have previously been shown to be effective with general-foot pain and disability in people with gout [64–66].

## Conclusion

This review aimed to evaluate and summarise the findings from existing literature which assessed the 1st MTP in people with gout. This review confirms the long-standing notion that acute 1st MTP arthritis is highly prevalent in people with gout and has a substantial impact on patient-reported outcomes related to pain and disability. Current research also suggests that the structure and function of the 1st MTP is impaired in people with gout. This review highlights the importance of clinical, laboratory and imaging findings related to the 1st MTP in the diagnosis of gout in clinical practice and underlines the need for interventions that specifically target improvements in structure, function and patient-reported outcomes related to the 1st MTP in people with gout.

## Abbreviation

1st MTP: First metatarsophalangeal joint
